# Ifosfamide-Induced Partial Arginine Vasopressin Resistance Responsive to Vasopressin/Desmopressin and Amiloride

**DOI:** 10.7759/cureus.83897

**Published:** 2025-05-11

**Authors:** Nicholas Ma, Haley Wilt, Patrick Donabedian, Shirley Kim

**Affiliations:** 1 Internal Medicine, University of Florida College of Medicine, Gainesville, USA

**Keywords:** arginine vasopressin resistance, doxorubicin and ifosfamide, hypernatremia polyuria diabetes insipidus, hypotonic polyuria, vasopressin/desmopressin

## Abstract

This is a case of partial arginine vasopressin resistance following the sixth cycle of doxorubicin-ifosfamide-mesna therapy for recurrent spindle cell sarcoma of the thigh. Polyuria and symptomatic hypernatremia started by the second day of the two-day chemotherapy cycle. The diagnosis was confirmed with serum and urine chemistry testing showing urine hypo-osmolality (161 mOsm/kg) with polyuria (4.8 L urine output) in 24 hours, serum hyperosmolality (355 mOsm/kg), and an elevated baseline plasma copeptin of 82 pmol/L. Treatment with intravenous vasopressin followed by amiloride and supraphysiologic doses of oral desmopressin improved symptomatic hypernatremia.

## Introduction

Arginine vasopressin disorder, previously known as diabetes insipidus, is a disorder that is characterized by the excretion of large amounts of dilute urine (>50 mL/kg/day) with associated polydipsia (>3 L/day) [1,2]. This disorder is caused by either arginine vasopressin deficiency (AVP-D, formerly central diabetes insipidus) due to pituitary dysfunction, or resistance (AVP-R, formerly nephrogenic diabetes insipidus) at target receptors. AVP-R can be partial or complete; it can result from either congenital mutation in AVP receptors or aquaporin channels, or acquired from various causes, including electrolyte abnormalities, sarcoidosis, or drug toxicity [1]. Acquired AVP-R treatment includes addressing the underlying cause, fluid management, correction of electrolyte abnormalities, and possible use of thiazide diuretics, which have been known to exert a paradoxical antidiuretic effect by decreasing sodium and chloride absorption in the distal tubule and thus increasing sodium and water absorption in the proximal tubule [1].

Ifosfamide is an alkylating chemotherapy agent often associated with proximal renal tubular toxicity, particularly Fanconi syndrome and AVP-R [3-7]. Chloroacetaldehyde, a metabolite of ifosfamide, has inhibitory effects on proximal tubular Na-K-ATPase activity [8,9]. It is also theorized that ifosfamide impairs urine concentration at the renal collecting duct via partial down-regulation of the basolateral AVPR2 [5]. This study reports the management of ifosfamide-induced AVP-R using supraphysiologic vasopressin (AVP)/desmopressin (DDAVP) and amiloride.

## Case presentation

A 42-year-old woman with a medical history significant for spindle cell sarcoma of the thigh developed polydipsia and polyuria on the second day of her sixth cycle of doxorubicin-ifosfamide-mesna, with a cumulative dose of 45 g/m^2^ ifosfamide. Her vitals and physical exam were unremarkable when admitted on the medicine service with co-management from hematology-oncology. However, she was quickly upgraded to the Medical Intensive Care Unit after becoming tremulous, agitated, and confused albeit without any focal neurologic deficits.

**Figure 1:**
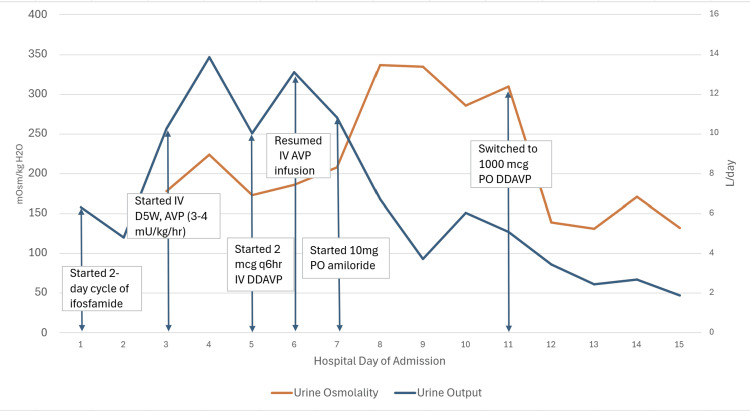
Response of urine osmolality and output in an AVP-R patient treated with AVP/DDAVP and amiloride AVP-R: Arginine vasopressin resistance, AVP: Vasopressin, DDAVP: Desmopressin

**Figure 2:**
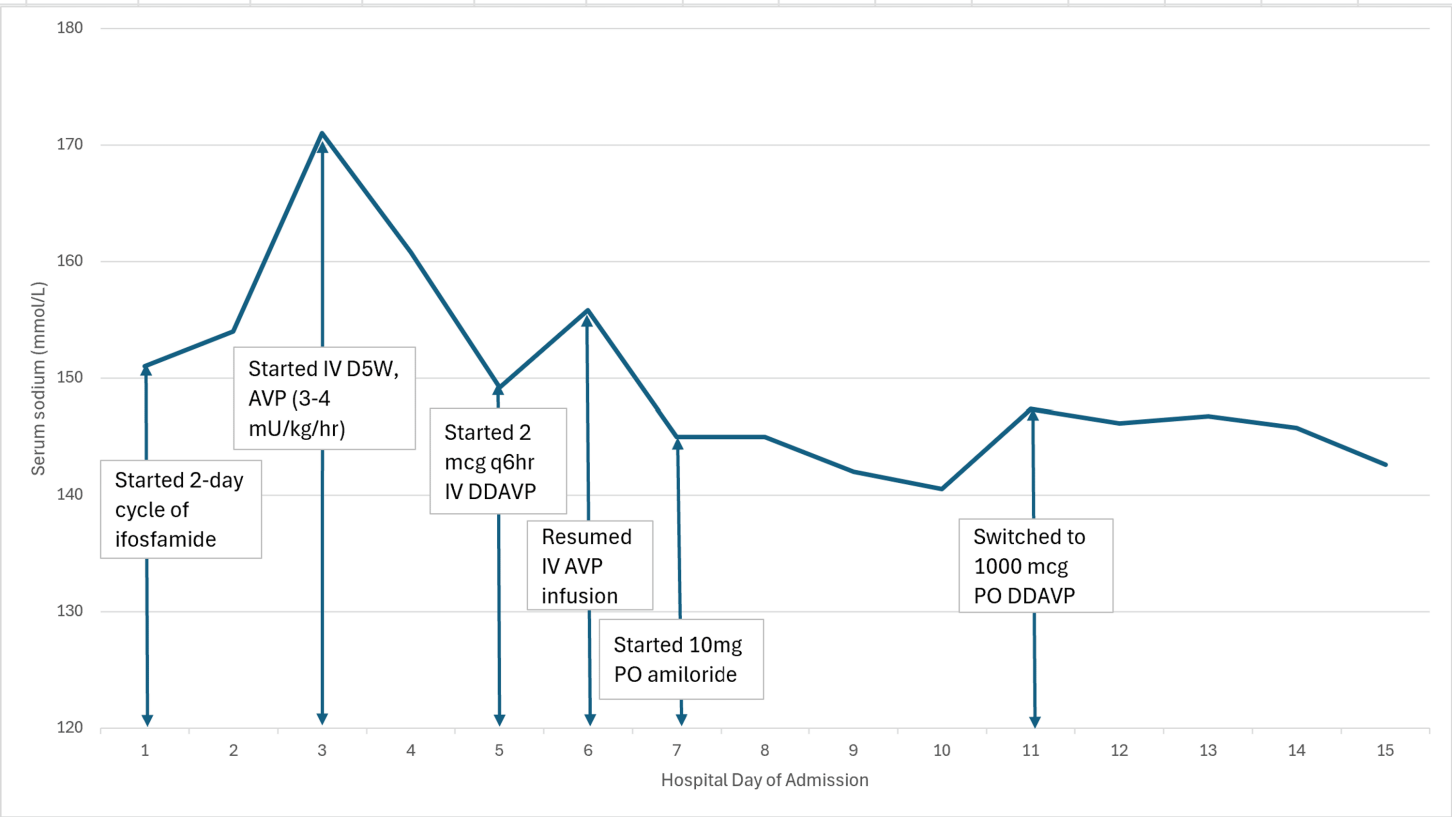
Response of serum sodium to AVP-R treatment AVP-R: Arginine vasopressin resistance

Initial laboratory studies were significant for serum sodium 155 mmol/L, potassium 3.3 mmol/L, and serum osmolality 355 mOsm/kg (Table 1). Urine studies showed urine osmolality 161 mOsm/kg, urine sodium 20 mmol/L, and urine phosphate <10 mg/dL, and urine pH 7.0 (Table 1); urinalysis was negative for blood, nitrites, leukocyte esterase, protein, and ketones, with rare bacteriuria and pyuria. From days 2-3, urine output increased from 4.8 to 10.25 L (Figure 1) and serum sodium increased from 155 to 171 mmol/L (Figure 2). Given her symptomatic hypernatremia (>147 mmol/L) and hypovolemia, baseline plasma copeptin level was indicated in lieu of water deprivation testing [2]. Baseline copeptin was 82 pmol/L (Table 1), with >21.4 pmol/L consistent with AVP-R [2,10].

**Table 1:** Patient's initial laboratory values upon hospitalization

Test	Patient value	Reference range
Sodium	155 mmol/L	136–145 mmol/L
Potassium	3.3 mmol/L	3.3–5.0 mmol/L
Bicarbonate	27 mmol/L	22–28 mmol/L
Creatinine	0.9 mg/dL	0.38–1.02 mg/dL
Phosphorus	3 mg/dL	2.7–4.5 mg/dL
Magnesium	2.4 mg/dL	1.5–2.8 mg/dL
Calcium	8.6 mg/dL	8.4–10.2 mg/dL
Serum osmolality	355 mOsm/kg	275–295 mOsmol/kg
Urine osmolality	161 mOsm/kg	50 to 1,100 mOsm/kg
Urine sodium	20 mmol/L	20–40 mmol/L
Urine phosphate	<10 mg/dL	<10 mg/dL
Urine pH	7.0	5.0-8.0
Copeptin	82 pmol/L	<13.1 pmol/L (non-water deprived adults); <15.2 pmol/L (water-deprived adults)

She received IV D5W at an infusion rate of 150 cc/hr to treat a 6.5L free water deficit with a goal of serum sodium 145 mmol/L. Endocrinology consultation recommended starting AVP intravenous infusion, up-titrating from 0.5 milliunits/kg/hr until achieving greater than 50% improvement of urine osmolality. Once she was on 3-4 milliunits/kg/hr of AVP from days 3-4, her urine osmolality increased from 161 to 287 mOsm/kg (Figure 1). The treatment was briefly converted to intravenous DDAVP, but intravenous AVP infusion was resumed and maintained for an additional five days due to polyuria recurrence, in addition to starting amiloride for borderline hypokalemia. Serum sodium and urine output and osmolality stabilized by day 10 (Figures 1, 2). Thereafter, AVP and amiloride were discontinued by discharge, and oral DDAVP and hydrochlorothiazide (given resolved hypokalemia risk) were started to maintain serum sodium and prevent polyuria.

## Discussion

In the process of assessing for AVP disease, our decision was influenced by the diagnostic accuracy of assessing baseline copeptin in lieu of performing a water deprivation test. Without prior water deprivation, a single baseline copeptin, using a cut-off of >21.4 pmol/L, has been found to have up to 100% sensitivity and specificity for diagnosing AVP-R in patients with serum hyperosmolality [10]. In contrast, indirect water deprivation has a lower diagnostic accuracy of 76.6% (sensitivity 86.4%, specificity 69.5%), since water diuresis of any cause will affect the renal medullary concentration gradient and thus down-regulate renal aquaporin channels [11]. Nonetheless, relying on a single biomarker for diagnosis is not as confirmatory as renal biopsy or broader biomarker investigations. With similar clinically unstable scenarios where diagnostic evaluation options are limited, establishing a more standardized copeptin threshold for distressed patients could improve diagnostic power.

Ifosfamide-induced AVP-R is a rare phenomenon, but previous cases involved comorbid Fanconi Syndrome and responsiveness to supraphysiologic DDAVP doses. In one case, AVP-R from a cumulative ifosfamide dose 10 g/m^2^ was treated by 2 mcg intravenous DDAVP, which resulted in immediate improvement of urine osmolality from 253 to 331 mOsm/kg over two hours; subsequent daily administration of 40 mcg intranasal DDAVP allowed for resolution of hypernatremia and polyuria by day 13 of hospitalization [3]. A similar case also used 20 mcg intranasal DDAVP every six hours with resolution of AVP-R after six days of therapy, increasing urine osmolality from 186 to over 350 mOsm/kg as well as decreasing urine output from 8.1 to 2-3 L [4]. A case of AVP-R following a cumulative ifosfamide dose of 7.5 g/m^2^ was treated with 40 mcg intravenous DDAVP every eight hours on day 2 of hospitalization, increased to 80 mcg every six to eight hours by day 4, and achieved an increase in urine osmolality up to 498 mOsm/kg with a sustained decrease in urine output 2-3 L/d by day 13 [5]. The acute response in urine osmolality to DDAVP in all of the above cases is consistent with partial AVP-R from ifosfamide, though resolution of electrolyte abnormalities and polyuria may require up to two weeks of treatment.

Compared to the literature, our case did not have confirmed Fanconi syndrome, given the lack of phosphaturia or aciduria (Table 1), but was responsive to AVP/DDAVP, suggesting partial rather than complete AVP-R. Difficulty with stabilizing urine output and concentration after converting to DDAVP therapy suggests significant resistance. This was overcome by administration of amiloride with AVP, which resulted in sustained improvement in polyuria, urine osmolality, hypernatremia, and overall symptomatic control. This supports prior cases showing the effectiveness of amiloride in treating ifosfamide-induced AVP-R [6] and lithium-induced partial AVP-R [12,13]. Nonetheless, the evolving clinical course and treatment process limit the ability to establish a direct causal response to the therapeutic interventions. Such demonstration may be established by starting treatment with AVP or DDAVP and a diuretic with paradoxical antidiuretic effects in AVP-R (such as amiloride or thiazide diuretics) for similar cases of AVP-R under controlled conditions.

## Conclusions

In summary, we present a case of ifosfamide-induced AVP-R, which was diagnosed with a single baseline copeptin and treated on a regimen consisting of supraphysiologic AVP/DDAVP and amiloride. The addition of a diuretic with supraphysiologic DDAVP can augment correction and maintenance of fluid and electrolyte balance in the setting of a relatively higher cumulative exposure to ifosfamide. Additional research could investigate standardized copeptin thresholds for diagnosis of AVP-R in similar cases of clinically distressed patients with polyuria and polydipsia. Further study of initial co-administration of supraphysiologic AVP or DDAVP and diuretic therapy upon AVP-R onset could also confirm efficacy of the therapy course as outlined in this study, which was only achieved after alterations to treatment with initial suboptimal control.
